# Interventricular Septal Involvement Is Associated with More Impaired Ventricular Function and Mechanics in Apical Hypertrophic Cardiomyopathy

**DOI:** 10.3390/jcdd11030074

**Published:** 2024-02-21

**Authors:** Christos G. Mihos, Tarec K. Elajami, Deepika Misra, Pranav Venkataraman, Nicholas Gosdenovich, Rafle Fernandez

**Affiliations:** 1 Echocardiography Laboratory, Columbia University Irving Medical Center, Division of Cardiology, Mount Sinai Heart Institute, Miami Beach, FL 33140, USA; rafle.fernandez@msmc.com; 2 Columbia University Irving Medical Center, Division of Cardiology, Mount Sinai Heart Institute, Miami Beach, FL 33140, USA; tarec.elajami@msmc.com (T.K.E.); deepika.misra@msmc.com (D.M.); pranav.venkataraman@msmc.com (P.V.); ngosd001@fiu.com (N.G.)

**Keywords:** apical hypertrophic cardiomyopathy, cardiac mechanics, global longitudinal strain, myocardial work, speckle-tracking echocardiography, strain echocardiography

## Abstract

Background: The interventricular septum has an important role in bi-ventricular performance. We hypothesized that septal involvement in apical hypertrophic cardiomyopathy (ApHCM-Mixed) adversely impacts ventricular structure and function when compared with isolated apical hypertrophy (ApHCM-Pure). Methods: A total of 72 patients (ApHCM-Mixed = 36, ApHCM-Pure = 36) with serial 2D and speckle-tracking echocardiographic analyses were identified. Ventricular function and mechanics were characterized by left (LV) and right (RV) ventricular global longitudinal strain (GLS), RV free wall strain, and LV myocardial work indices, and clinical events were adjudicated. Results: Clinical characteristics were similar between groups (mean age, 66 ± 15 years; 49% female; LV ejection fraction, 68 ± 11%). The ApHCM-Mixed group had larger LV mass indexes (141 ± 39 vs. 111 ± 30 g/m^2^, *p* < 0.001), worse LV (−9.6 ± 3.1 vs. −14.4 ± 3.4%, *p* < 0.001) and RV GLS (−14.3 ± 6.7 vs. −19.2 ± 5.2%, *p* = 0.001), impaired RV free wall strain (−18.5 ± 7.4 vs. −22.4 ± 6.3%, *p* = 0.02), and lower LV myocardial work indices including global work index (938 ± 306 vs. 1272 ± 339 mmHg%, *p* < 0.001), when compared with the ApHCM-Pure group. At a mean follow-up of 3.9 years, these differences all persisted. Five deaths were observed, all occurring in the ApHCM-Mixed group (14% vs. 0, *p* = 0.05), and with four being cardiac-related. This subgroup had a mean LV ejection fraction of 63%, LV GLS of −8.7%, an LV global work index of 875 mmHg%, and RV free wall strain of −15.9%, indicating significant subclinical bi-ventricular dysfunction. Conclusions: ApHCM-Mixed represents a distinct morphology in hypertrophic cardiomyopathy associated with more impaired ventricular function and mechanics when compared with ApHCM-Pure.

## 1. Introduction

Apical hypertrophic cardiomyopathy (ApHCM) is characterized by increased apical left ventricular (LV) wall thickness ≥ 15 mm with an ‘ace of spades’ morphology in the absence of abnormal loading conditions or identifiable causes [1]. It accounts for approximately 20% of all hypertrophic cardiomyopathy cases, may be sporadic or inherited due to myofilament gene mutations, and has contemporaneously been shown to confer important cardiovascular morbidity [2,3,4,5,6]. Speckle-tracking strain mechanics reveal that despite a generally preserved or even hyperdynamic LV ejection fraction, patients with ApHCM have a progressive impairment of LV global longitudinal strain (GLS) and myocardial energetics, which most commonly manifests as heart failure with a preserved ejection fraction [7,8].

The ApHCM variant presents as two morphologic phenotypes: a “pure” form defined as hypertrophy confined to the LV apex distal to the papillary muscles (ApHCM-Pure), and a “mixed” form defined as hypertrophy involving the apex and extending to include the interventricular septum (ApHCM-Mixed) [2,3]. This phenotypic distinction is salient given the importance of the interventricular septum to bi-ventricular mechanical performance and efficiency [9,10]. Despite the clinical significance of ApHCM within the spectrum of cardiomyopathic disorders, there are limited data comparing ApHCM-Pure and ApHCM-Mixed [11,12,13]. A single study signaled that ApHCM-Mixed may be independently associated with a greater than 3-fold increased risk of cardiovascular morbidity, which was defined as hospitalization for syncope, congestive heart failure, nonfatal arrhythmia, stroke, or myocardial infarction [11]. However, whether this is related to the presence of interventricular septal pathology, and the knowledge gaps underlying the physiologic substrate in these patients, remain unresolved.

The assessment of ventricular GLS and myocardial energetics, the latter assessed as myocardial work and incorporating LV afterload, allows for the detailed assessment of cardiac functional mechanics, energy consumption, and subclinical dysfunction. This is particularly useful in diseases such as ApHCM where the LV ejection fraction is typically normal or hyperdynamic. The aim of this retrospective cohort study of patients with ApHCM referred for echocardiography is to (1) test the hypothesis that when compared with ApHCM-Pure, the ApHCM-Mixed phenotype is associated with more advanced LV functional remodeling as measured by GLS and myocardial work indices using 2D speckle-tracking echocardiography; and (2) to compare their natural history in terms of follow-up echocardiographic and clinical outcomes assessments.

## 2. Materials and Methods

### 2.1. Patient Selection and Definitions

The study protocol was drafted in accordance with the 2013 updated Declaration of Helsinki ethical guidelines and was approved by the Institutional Review Board at Mount Sinai Medical Center/Mount Sinai Heart Institute, Miami Beach, FL, USA. Patient consent was waived by the institutional review board due to the retrospective nature of the study, and all of the analyses were performed on historical data which in no way impacted the patients’ rights or medical care. Our institutional echocardiography digital database was retrospectively searched to identify ApHCM patients referred for echocardiography between January 2005 and January 2021, with at least one follow-up echocardiogram required for inclusion and comparative review. Review of the institutional electronic medical records was performed to document demographics, clinical risk factors, and laboratory values. Patients with the following conditions were excluded: (1) untreated or uncontrolled hypertension; (2) hypertensive heart disease; (3); infiltrative cardiomyopathy; (4) phenocopy conditions (i.e., Anderson–Fabry disease, Danon disease, Friedrich’s ataxia). The follow-up clinical outcomes adjudicated were cardiovascular mortality, myocardial infarction, sudden cardiac death, cerebrovascular accident, any cardiovascular hospitalization, heart failure hospitalization, and all-cause mortality. The follow-up of each individual patient was determined as the latest presentation to our institution for clinical care, or at an external institution as abstracted from shared electronic health records, as available.

**Figure 1:**
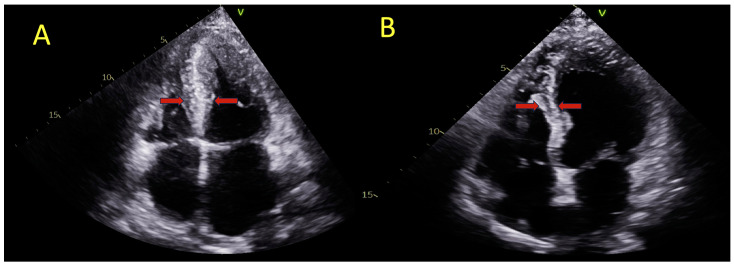
Two-dimensional echocardiographic four-chamber view at end-diastole depicting a patient with apical hypertrophic cardiomyopathy and septal involvement (**A**), and a patient with pure apical hypertrophy (**B**). The red arrows highlight the interventricular septal thickness.

ApHCM was defined as LV apical wall thickness ≥15 mm distal to the insertion points of the papillary muscles, which is not explained by loading conditions or secondary causes [1]. The patients were stratified according to two phenotypes: ApHCM-Pure was defined as hypertrophy confined to the LV apex distal to the papillary muscles, and ApHCM-Mixed was defined as hypertrophy involving the LV apex and extending to include the interventricular septum [2,3] (Figure 1). Apical aneurysm was defined as thinned and dyskinetic apical myocardial segments with a distinct neck [14]. Obstruction was defined as a peak instantaneous intra-cavitary or left ventricular outflow tract (LVOT) pressure gradient of ≥30 mmHg at rest or with provocative maneuvers [1].

### 2.2. Two-Dimensional and Speckle-Tracking Echocardiography

The transthoracic echocardiograms were performed using a GE E9, E95, or S70 cardiovascular ultrasound system (General Electric Healthcare, Waukesha, WI, USA). The American Society of Echocardiography chamber quantification guidelines, and recommendations for the evaluation of LV diastolic function, were applied to assess cardiac geometry, systolic function, and diastology [15,16]. The maximal LV septal and posterior wall thickness and mass were assessed in the parasternal long-axis view at end-diastole. The three standard apical views, and a cross-sectional parasternal short-axis view distal to the papillary muscle insertions, were utilized to assess maximal apical wall thickness. The assessment of mitral valve anatomy, and the presence and grading of regurgitant lesions, was performed in a multi-parametric manner according to the native valvular regurgitation guidelines [17].

All speckle-tracking strain echocardiography was performed using the GE EchoPAC Automated Function Imaging and Q-Analysis software (General Electric Healthcare, Waukesha, WI, USA) according to inter-societal consensus statements on cardiac mechanics quantitation [18,19]. Peak GLS was measured and averaged in the apical four-, three- and two-chamber views. Myocardial work was calculated by integrating longitudinal strain, and LV afterload as estimated by the brachial artery cuff blood pressure, to generate an LV pressure–strain loop with adjusted ejection and isovolumetric periods [20,21]. The four myocardial work parameters measured were defined as follows with cited normal reference values: (1) global work index (>1576 mmHg%)—area of the LV pressure–strain loop between mitral valve opening and closure representing the total LV work performed during systolic ejection and isovolumic relaxation; (2) global constructive work (>1708 mmHg%)—segmental shortening during systole plus lengthening during isovolumic relaxation; (3) global wasted work (<159 mmHg%)—segmental lengthening during systole plus shortening during isovolumic relaxation; and (4) work efficiency (>93%)—calculated by the following equation: constructive work/(constructive work + wasted work) × 100 [22]. Right ventricular (RV) speckle-tracking mechanics were assessed from an apical RV-focused view as GLS which includes the free wall and interventricular septum and free wall strain only. All echocardiograms used in the study were analyzed by two level-III board certified echocardiographers (C.G.M., R.F.) with expertise in speckle-tracking echocardiography.

### 2.3. Statistical Analyses

The IBM SPSS Statistics version 21 software (IBM Corporation, Armonk, NY, USA) was utilized in the statistical analyses. Categorical variables were expressed as numbers (frequency percentages), while continuous variables were expressed as means ± standard or medians (interquartile ranges), dependent upon their normality. In the event of insufficient image quality for speckle-tracking mechanics, the well-described multiple imputation technique was applied. Of note, it is estimated that this was observed in <15% of the patients [23]. Intergroup continuous variables were compared using an independent *t*-test, while a paired *t*-test was applied for intragroup repeated measures. A chi-square or Fisher’s exact test was applied in the comparison of categorical variables, as appropriate. A *p*-value < 0.05 was considered statistically significant.

## 3. Results

### 3.1. Patient Demographics and Clinical Characteristics

A total of 72 patients were identified, with 36 in each of the groups. The mean age of the cohort was 66 ± 15 years, 35 (49%) were female, and 1 (3%) had a genetically confirmed family history of HCM. The most common co-morbidities were hypertension (89%), atrial fibrillation (33%), and coronary artery disease (29%), with 22 (31%) patients having NYHA functional class ≥ II heart failure symptoms. There was no difference in demographics or clinical characteristics between the ApHCM-Pure and ApHCM-Mixed groups (Table 1). The median follow-up was 3.9 years (interquartile range, 1.6–9.2).

### 3.2. Two-Dimensional and Speckle-Tracking Echocardiographic Analyses

**Figure 2:**
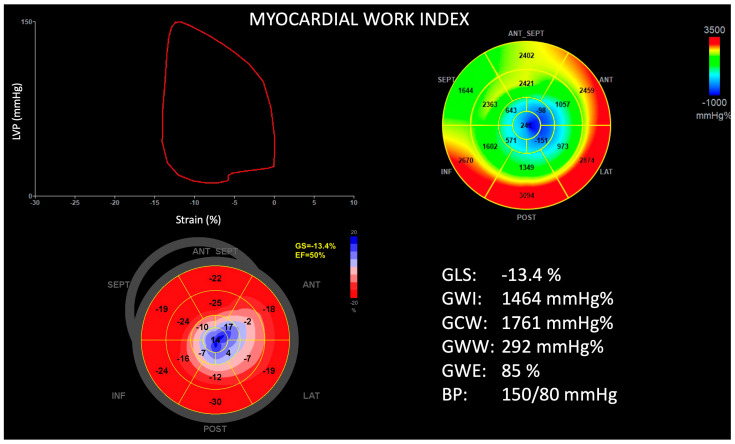
Example of left ventricular global longitudinal strain and myocardial work in a patient with pure apical hypertrophic cardiomyopathy. Top left, pressure–strain loop. Top right, myocardial work polar map. Bottom left, peak systolic strain polar map. Bottom right, summary of measurements. ANT = anterior; ANT SEPT = anteroseptal; BP = blood pressure; EF = ejection fraction; GCW = global constructive work; GLS = global longitudinal strain; GS = global strain; GWE = global work efficiency; GWI = global work index; GWW = global wasted work; INF = inferior; LAT = lateral; LVP = left ventricular pressure; POST = posterior; SEPT = septal.

**Figure 3:**
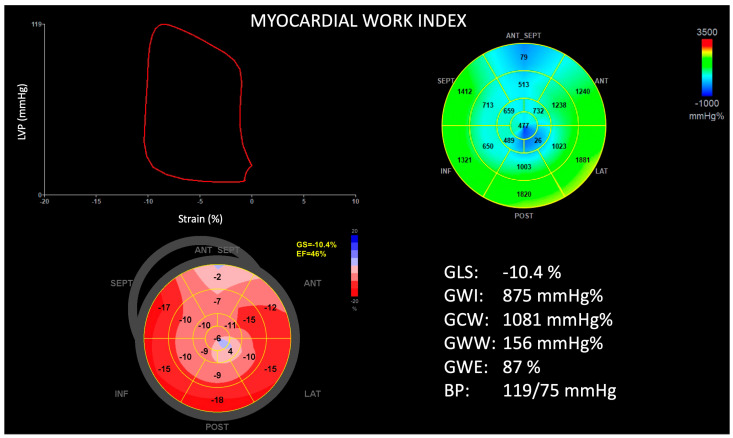
Example of left ventricular global longitudinal strain and myocardial work in a patient with mixed apical hypertrophic cardiomyopathy. Top left, pressure–strain loop. Top right, myocardial work polar map. Bottom left, peak systolic strain polar map. Bottom right, summary of measurements. ANT = anterior; ANT SEPT = anteroseptal; BP = blood pressure; EF = ejection fraction; GCW = global constructive work; GLS = global longitudinal strain; GS = global strain; GWE = global work efficiency; GWI = global work index; GWW = global wasted work; INF = inferior; LAT = lateral; LVP = left ventricular pressure; POST = posterior; SEPT = septal.

The mean LV ejection fraction was measured as 68 ± 11% and did not differ between groups. Patients with ApHCM-Mixed had a larger LV mass index (141 ± 39 vs. 111 ± 30 g/m^2^, *p* < 0.001), thicker septal (1.8 ± 0.2 vs. 1.2 ± 0.2 mm, *p* < 0.001) and apical walls (1.9 ± 0.3 vs. 1.8 ± 0.3 mm, *p* = 0.05), and a greater prevalence of RV hypertrophy (33 vs. 14%, *p* = 0.05) when compared with ApHCM-Pure. Additionally, ApHCM-Mixed patients were characterized by more impaired LV GLS (−9.6 ± 3.1 vs. −14.4 ± 3.4%, *p* < 0.001), a lower global work index (938 ± 306 vs. 1272 ± 339 mmHg%, *p* < 0.001), constructive work (1211 ± 383 vs. 1654 ± 453 mmHg%, *p* < 0.001), and work efficiency (79 ± 8 vs. 85 ± 6%, *p* = 0.001), and greater wasted work (288 ± 178 vs. 208 ± 153 mmHg%, *p* = 0.05), when compared with ApHCM-Pure (Figure 2 and Figure 3). The RV mechanics, as assessed by RV GLS (−14.3 ± 6.7 vs. −19.2 ± 5.2%, *p* = 0.001) and free wall strain (−18.5 ± 7.4 vs. −22.4 ± 6.3%, *p* = 0.02), were also observed to be worse in the ApHCM-Mixed group. Of note, there were seven (9%) patients who had evidence of obstructive LV physiology (ApHCM-Pure = three, ApHCM-Mixed = four). In the ApHCM-Pure group, intra-cavitary obstruction was present in two patients and LVOT obstruction in one patient. In the ApHCM-Mixed group, there were three patients with LVOT obstruction and one with intra-cavitary obstruction. The average peak systolic pressure gradient in these patients was 54 ± 15 mmHg and did not differ between groups.

At follow-up, there was a progressive decline in LV performance in the ApHCM-Pure group, although a greater degree of impairment persisted in the ApHCM-Mixed group upon intergroup comparison. This included the measures of LV GLS (−9.5 ± 2.9 vs. −12.0 ± 3.2%, *p* = 0.001), global work index (891 ± 345 vs. 1086 ± 316 mmHg%, *p* = 0.02), and global constructive work (1180 ± 370 vs. 1444 ± 329 mmHg%, *p* = 0.002). Additionally, LV global wasted work increased and work efficiency decreased in the ApHCM-Pure group, with no demonstrable difference when compared with the ApHCM-Mixed group. In regard to RV mechanics, the RV GLS remained more impaired in the ApHCM-Mixed patients (−15.5 ± 4.3 vs. 17.9 ± 4.4%, *p* = 0.02), with an attenuated difference between groups in free wall strain. Finally, ApHCM-Mixed patients had a higher E/e’ ratio when compared with ApHCM-Pure patients, which is suggestive of worse diastolic function and increased LV filling pressure (14 ± 4 vs. 17 ± 9, *p* = 0.06) (Table 2).

### 3.3. Clinical Outcomes

All-cause mortality occurred in five patients, all in the ApHCM-Mixed group (14% vs. 0, *p* = 0.05), and with four being cardiovascular mortalities (Table 3). This subgroup had a mean LV ejection fraction of 63 ± 15%, LV GLS of −8.7 ± 5.2%, LV global work index of 875 ± 412 mmHg%, LV filling pressure of 14 ± 4, and RV free wall strain of −15.9 ± 8.6% (Table 4). Of the four patients with a cardiovascular mortality, there were two sudden cardiac deaths, one case of end-stage heart failure, and one fatal myocardial infarction. Additional adverse events included 2 (3%) sudden deaths, 6 (8%) myocardial infarctions, 7 (10%) cerebrovascular accidents, 28 (39%) cardiovascular-related hospitalizations, and 14 (19%) heart failure hospitalizations, with no difference in prevalence between the ApHCM groups in these outcomes.

## 4. Discussion

In this retrospective study of 72 ApHCM patients stratified by involvement of the interventricular septum versus pure apical hypertrophy, the salient findings can be summarized as follows: (1) the mean age was 66 years and approximately half were female, with no demonstrable difference in demographics or clinical risk factors between ApHCM-Mixed and ApHCM-Pure patients; (2) despite a similar LV ejection fraction, patients with ApHCM-Mixed had more impaired LV GLS, lower global work indices, and more wasted work, as well as worse RV GLS and free wall strain; (3) a progressive decline in LV performance in the ApHCM-Pure group was observed at the 3.9-year follow-up, although a greater degree of impairment persisted in ApHCM-Mixed on comparison; and (4) all-cause mortality occurred in five patients exclusively with ApHCM-Mixed and significant subclinical bi-ventricular dysfunction, with four deaths being cardiovascular-related.

Decreased ventricular mechanics and performance characterize hypertrophic cardiomyopathy, with an LV global constructive work ≥ 1730 mmHg% having been identified as a threshold for better event-free survival, and GLS > −17% being related to the extent of late gadolinium enhancement on cardiac magnetic resonance imaging [7,24]. More specifically, when compared with non-ApHCM phenotypes, patients with ApHCM have been shown to have greater and progressive LV impairment [7,8]. In a study comparing 48 ApHCM versus 69 non-ApHCM patients, the median LV GLS (−11 vs. −18%), global work index (966 vs. 1803 mmHg%), and global constructive work (1050 vs. 1998 mmHg%) (all *p* < 0.001) were significantly attenuated amongst the ApHCM group, which is consistent with marked subclinical LV dysfunction and abnormal myocardial energy consumption [7]. Global wasted work > 186 mmHg% has also been shown to predict adverse cardiovascular outcomes in ApHCM, with sensitivity of 93%. The present study expanded upon these concepts and showed that amongst patients with ApHCM, involvement of the interventricular septum represents a distinct morphology along the disease spectrum with more advanced bi-ventricular dysfunction than patients with ApHCM-Pure. Given these findings and the observation of all five deaths having occurred in the ApHCM-Mixed group, there is merit in conducting larger longitudinal and multi-center registry studies. Furthermore, if these trends were to be confirmed in larger cohorts, the clinical approach to ApHCM-Mixed patients may require important reappraisal both in terms of sudden death prevention and heart failure therapy.

Normal function of the interventricular septum is paramount to optimal bi-ventricular performance by contributing to LV and RV electromechanical association and systolic and diastolic function [9,10]. More specifically, the shared septum results in ventricular interdependence and is composed of oblique helical myocardial fibers that produce longitudinal shortening and lengthening, which is measured as strain. It has been shown that these mechanics are responsible for upwards of 80% of the RV systolic ejection, underscoring the importance of septal anatomy and function [9,10,25]. In the present study, in addition to more impaired LV GLS and myocardial work indices in patients with ApHCM-Mixed versus ApHCM-Pure, we observed abnormal RV GLS and free wall strain indicative of dysfunction in the RV mechanics. For patients with HCM, RV GLS is strongly associated with exercise functional capacity, and free wall strain > −20% has been identified as a predictor of adverse outcomes at follow-up [26,27]. Additionally, the ApHCM-Mixed group had more RV involvement in the form of RV hypertrophy when compared with the ApHCM-Pure group. In patients with HCM, RV hypertrophy has been shown to be associated with more markedly impaired bi-ventricular mechanics and a higher prevalence of heart failure hospitalizations than those without RV hypertrophy [28]. Thus, the careful visualization of the right heart and the detailed assessment of RV remodeling and mechanics should be performed in all HCM patients.

At mid-term follow-up, we observed a progressive decline in LV mechanics in the ApHCM-Pure group, although a greater degree of impairment persisted in ApHCM-Mixed patients upon comparison. This adds to the characterization of ApHCM as a progressive cardiomyopathy, and, with the aforementioned discussion, suggests that ApHCM-Mixed represents a distinct subtype with more advanced ventricular dysfunction and concerns regarding observed all-cause mortality, which requires further investigation [7,8,11,12,13]. Previously identified risk factors for poor LV GLS in ApHCM include atrial fibrillation, mitral annular e’ velocity, and glomerular filtration rate, and RV hypertrophy for RV GLS [8,27]. Given the cardiovascular morbidity associated with ApHCM, these data, when taken together, allow for the growth of tailored patient risk stratification and therapeutic planning.

As with all echocardiographic examinations, careful scanning with proper techniques and imaging windows is paramount to identifying all pertinent pathologies in patients with ApHCM. This includes non-foreshortened apical windows, the use of intravenous ultrasound enhancing agents, and the assessment of the interventricular septum from parasternal long-axis, short-axis, and apical views. The latter point was applied in our study and is salient in that the septum may be asymmetrically thickened anteriorly or inferiorly, and reliance on only a single plane may lead to phenotypic mischaracterization. Additional methodological strengths include the strict inclusion and exclusion criteria and the measurement of myocardial work in order to take into account the effect of LV afterload on cardiac mechanics. These aforementioned factors supported robust study data and analytics.

There are limitations that are important to keep in consideration when interpreting the study results. First, this was a retrospective study with a small sample size, which limits its statistical power and predisposes it to type II statistical error. For this reason, the clinical outcome of all-cause mortality is interpreted cautiously, and competing causes for death are not adjudicated. Second, a total of 98 patients with ApHCM are present in our institutional echocardiography digital database, of which, 26 did not have a follow-up echocardiogram for review and were excluded from the study analyses. This represents a form of attrition bias and is an uncontrollable confounder. Third, individual segmental LV strain analysis was not available in the present study, which may be used to differentiate the extent of impairment in and between LV territories. Nevertheless, task force criteria recommend the use of LV GLS over segmental strain analyses, given the measurement heterogeneity of single segmental strains and the plethora of published outcome data on LV GLS [18,19]. Fourth, as previously discussed, a small minority of patients were observed to have either intra-cavitary or LVOT dynamic obstruction, which does impart increased afterload on the LV walls. This was not accounted for in the pressure–strain loop assessment and may have meant that myocardial work was underestimated. While some investigators suggest adding the peak or mean dynamic pressure gradient to the brachial cuff pressure used in myocardial work estimation, others have shown good accuracy and predictive value without accounting for the additional afterload, and this remains unsettled in the published literature [29,30]. Fifth, cardiac magnetic resonance imaging was not routinely performed or available for review, which is a modality important in HCM for myocardial tissue characterization and the assessment of late gadolinium enhancement. The causes for this are our tertiary referral center services and our expansive patient population of underserved individuals who do not have access to these advanced and at times costly tests. For similar reasons, genetic testing was only available in a few patients and extended family members, which almost certainly confers an underestimation of the familial prevalence of HCM in our cohort. Sixth, intravenous echocardiographic contrast to enhance the endocardial border was not routinely utilized. This may have resulted in the underestimation of the prevalence of LV apical aneurysms in ApHCM, which are associated with significant morbidity. Seventh, owing to the retrospective analysis of an established database, the inter- and intraobsever variability was not assessed. Eighth, patients with ApHCM had a higher prevalence of calcium-channel blocker use. It is not known if the calcium channel-blockers were non-dihydropyridine or dihydropyridine agents, which are preferentially used for heart control versus peripheral vasodilatation and may influence measures of ventricular mechanics. Thus, our results are best interpreted as hypothesis-generating, require external validation from larger dedicated HCM program cohorts and multi-center registries, and should not be liberally applied to phenotypes outside of ApHCM.

In conclusion, ApHCM-Mixed represents a distinct phenotype in HCM associated with more impaired bi-ventricular function and mechanics as compared with ApHCM-Pure. Continued deterioration in LV function was observed over the mid-term follow-up, with all mortalities occurring amongst the ApHCM-Mixed patients. These findings provide a novel perspective on ApHCM and suggest that clinical decision making, risk stratification, and prognosis should be individualized according to the underlying phenotype. Whether specific medical therapy, or percutaneous and surgical interventions, are beneficial in this population remains the focus of ongoing investigation.

## Figures and Tables

**Table 1:** Demographics and clinical characteristics of patients with pure versus mixed apical hypertrophic cardiomyopathy phenotype.

Variable	Apical-Pure N = 36	Apical-Mixed N = 36	*p*-Value
Age	65 ± 17	67 ± 13	0.66
Female	21 (58%)	14 (39%)	0.1
Body surface area (m^2^)	1.82 ± 0.26	1.92 ± 0.23	0.08
Heart rate (beats/minute)	69 ± 10	75 ± 18	0.08
Systolic blood pressure (mmHg)	128 ± 17	133 ± 20	0.24
Diastolic blood pressure (mmHg)	70 ± 12	75 ± 11	0.11
Serum creatinine (mg/dL)	1.11 ± 0.49	1.12 ± 0.66	0.96
African American	7 (19%)	6 (17%)	0.76
Smoking	8 (22%)	13 (36%)	0.2
Family history of hypertrophic cardiomyopathy	0	1 (3%)	1
Clinical signs and symptoms			
Angina	14 (39%)	18 (50%)	0.34
Dyspnea	11 (31%)	10 (28%)	0.8
Palpitations	10 (28%)	9 (25%)	0.79
Syncope	7 (19%)	3 (8%)	0.31
Non-sustained ventricular tachycardia	6 (17%)	2 (6%)	0.26
Hypertension	31 (86%)	33 (92%)	0.45
Diabetes mellitus	11 (31%)	9 (25%)	0.6
Coronary artery disease	9 (25%)	12 (33%)	0.44
Congestive heart failure	3 (8%)	8 (22%)	0.19
New York Heart Association functional class ≥ II	11 (31%)	11 (31%)	1
Cerebrovascular accident	3 (8%)	7 (19%)	0.31
Atrial fibrillation	9 (25%)	15 (42%)	0.13
Implantable cardioverter defibrillator	1 (3%)	3 (8%)	0.61
Medications			
Aspirin	15 (42%)	22 (61%)	0.1
ACEi/angiotensin receptor blocker	21 (58%)	14 (39%)	0.1
Beta-blocker	20 (56%)	24 (67%)	0.33
Calcium-channel blocker	8 (22%)	17 (47%)	0.03
Direct oral anticoagulant	8 (22%)	8 (22%)	1
Diuretics	8 (22%)	11 (31%)	0.42
P2Y12 inhibitor	6 (17%)	6 (17%)	1
Statin	20 (56%)	23 (64%)	0.47

ACEi = angiotensin converting enzyme inhibitor.

**Table 2:** Two-dimensional and speckle-tracking echocardiography in patients with pure versus mixed apical hypertrophic cardiomyopathy phenotype.

Variable						
Left Ventricular Structure and Function	ApHCM-Pure N = 36	ApHCM-Mixed N = 36	*p*-Value	ApHCM-Pure N = 36	ApHCM-Mixed N = 36	*p*-Value
LV ejection fraction (%)	67 ± 10	69 ± 12	0.44	63 ± 10	65 ± 19	0.54
LV internal diastolic diameter index (mm/m^2^)	25 ± 3	23 ± 4	0.01	25 ± 4	23 ± 3	0.05
LV internal systolic diameter index (mm/m^2^)	15 ± 4	14 ± 4	0.26	16 ± 4	14 ± 4	0.04
LV mass index (g/m^2^)	111 ± 30	141 ± 39	<0.001	110 ± 28	144 ± 38	<0.001
Septal wall thickness (mm)	1.2 ± 0.2 *	1.8 ± 0.2	<0.001	1.3 ± 0.2 *	1.8 ± 0.3	<0.001
Posterior wall thickness (mm)	1.1 ± 0.2	1.2 ± 0.2	0.08	1.1 ± 0.2	1.2 ± 0.2	0.18
Apical wall thickness (mm)	1.8 ± 0.3	1.9 ± 0.3	0.05	1.8 ± 0.3	2.0 ± 0.4	0.12
Relative wall thickness	0.51 ± 0.1	0.56 ± 0.1	0.06	0.52 ± 0.1	0.55 ± 0.1	0.23
Left ventricular apical aneurysm	3 (8%)	4 (11%)	1	4 (11%)	5 (14%)	0.72
Left ventricular mechanics						
Global longitudinal strain (%)	−14.4 ± 3.4 ^†^	−9.6 ± 3.1	<0.001	−12.0 ± 3.2 ^†^	−9.5 ± 2.9	0.001
Global work index (mmHg%)	1272 ± 339 ^†^	938 ± 306	<0.001	1086 ± 316 ^†^	891 ± 345	0.02
Global constructive work (mmHg%)	1654 ± 453 ^†^	1211 ± 383	<0.001	1444 ± 329 ^†^	1180 ± 370	0.002
Global wasted work (mmHg%)	208 ± 153 *	288 ± 178 ^#^	0.05	262 ± 136 *	224 ± 86 ^#^	0.17
Global work efficiency (%)	85 ± 6 *	79 ± 8	0.001	82 ± 6 *	80 ± 8	0.28
Left ventricular diastology						
Peak transmitral E-wave velocity (m/s)	0.79 ± 0.21	0.77 ± 0.24	0.7	0.78 ± 0.18	0.82 ± 0.25	0.48
Average mitral annular velocity (m/s)	0.07 ± 0.02 ^†^	0.06 ± 0.02	0.22	0.06 ± 0.01 ^†^	0.05 ± 0.02	0.26
Average E/e’ ratio	12 ± 3 ^†^	14 ± 6 ^#^	0.25	14 ± 4 ^†^	17 ± 9 ^#^	0.06
Right ventricular structure and function						
Right ventricular basal diameter (mm)	33 ± 5	33 ± 4	0.74	33 ± 6	34 ± 6	0.54
Tricuspid annular plane systole excursion (mm)	18 ± 3 ^†^	16 ± 3 ^‡^	0.15	15 ± 4 ^†^	14 ± 4 ^‡^	0.55
Right ventricular hypertrophy	5 (14%)	12 (33%)	0.05			
Right ventricular systolic pressure (mmHg)	32 ± 13	34 ± 11	0.56	35 ± 14	35 ± 16	1
Right ventricular mechanics						
Global longitudinal strain (%)	−19.2 ± 5.2	−14.3 ± 6.7	0.001	−17.9 ± 4.4	−15.5 ± 4.3	0.02
Free wall strain (%)	−22.4 ± 6.3	−18.5 ± 7.4	0.02	−21.7 ± 4.9	−19.5 ± 5.5	0.08
Left atrial volume index (mL/m^2^)	34 ± 12	39 ± 12	0.13	38 ± 14	39 ± 12	0.64
Mitral valve						
Systolic anterior motion	3 (8%)	6 (17%)	0.29	2 (6%)	4 (11%)	0.39
Moderate or severe mitral regurgitation	3 (8%)	2 (6%)	0.64	6 (17%)	2 (6%)	0.26

LV = left ventricle. * *p* < 0.05, ApHCM-Pure intragroup baseline versus follow-up repeated-measure analysis. ^#^ *p* < 0.05, ApHCM-Mixed intragroup baseline versus follow-up repeated-measure analysis ^†^ *p* < 0.01, ApHCM-Pure intragroup baseline versus follow-up repeated-measure analysis. ^‡^ *p* < 0.01, ApHCM-Mixed intragroup baseline versus follow-up repeated-measure analysis. Right ventricular systolic pressure was available in 27 ApHCM-Pure and 26 ApHCM-Mixed patients.

**Table 3:** Clinical follow-up outcomes of patients with pure versus mixed apical hypertrophic cardiomyopathy phenotype.

Variable	ApHCM-Pure N = 36	ApHCM-Mixed N = 36	*p*-Value
All-cause mortality	0	5 (14%)	0.05
Sudden death	0	2 (6%)	0.15
Myocardial infarction	2 (6%)	4 (11%)	0.39
Cerebrovascular accident	3 (8%)	4 (11%)	0.69
Any cardiovascular hospitalization	14 (39%)	14 (39%)	1
Heart failure hospitalization	6 (17%)	8 (22%)	0.55

**Table 4:** Clinical and imaging characteristics of five patients with mixed apical hypertrophic cardiomyopathy who met the outcome of all-cause mortality.

N	Gender	Age (Years)	CHF	CAD	AF	LVEF (%)	IVS (mm)	Apex (mm)	LVEDDi (mm/m^2^)	E/e’	GLS (%)	GWI (mmHg%)	RVFWS (%)
1	F	75	1	0	1	37	17	23	23	16	−3.6	329	−2.7
2	M	72	0	1	0	61	15	15	22	8	−13.2	1167	−23.9
3	M	69	0	0	1	69	15	15	19	10	−15.3	1405	−23.5
4	M	81	1	0	1	76	15	15	20	15	−5.6	1006	−8.7
5	F	86	0	1	0	70	23	23	19	19	−5.9	468	−20.7

AF = atrial fibrillation; CAD = coronary artery disease; CHF = congestive heart failure; GLS = global longitudinal strain; GWI = global work index; IVS = interventricular septal thickness; LVEDDi = left ventricular diastolic diameter index; LVEF = left ventricular ejection fraction; N = number; RVFWS = right ventricular free wall values.

## Data Availability

Due to institutional review board and ethical regulations, all publicly available data are contained within the article.
